# Complete Genome of the *Xanthomonas euvesicatoria* Specific Bacteriophage KΦ1, Its Survival and Potential in Control of Pepper Bacterial Spot

**DOI:** 10.3389/fmicb.2018.02021

**Published:** 2018-08-29

**Authors:** Katarina Gašić, Nemanja Kuzmanović, Milan Ivanović, Anđelka Prokić, Milan Šević, Aleksa Obradović

**Affiliations:** ^1^Institute for Plant Protection and Environment (IZBIS), Belgrade, Serbia; ^2^Institute for Epidemiology and Pathogen Diagnostics, Federal Research Centre for Cultivated Plants, Julius Kühn-Institut, Braunschweig, Germany; ^3^Department of Plant Pathology, Faculty of Agriculture, University of Belgrade, Belgrade, Serbia; ^4^Institute of Vegetable Crops Ltd., Smederevska Palanka, Serbia

**Keywords:** *Xanthomonas euvesicatoria*, bacteriophage, genome analysis, survival, phage therapy

## Abstract

*Xanthomonas euvesicatoria* phage KΦ1, a member of *Myoviridae* family, was isolated from the rhizosphere of pepper plants showing symptoms of bacterial spot. The phage strain expressed antibacterial activity to all *X. euvesicatoria* strains tested and did not lyse other *Xanthomonas* spp., nor other less related bacterial species. The genome of KΦ1 is double-stranded DNA of 46.077 bp including 66 open reading frames and an average GC content of 62.9%, representing the first complete genome sequence published for a phage infecting xanthomonads associated with pepper or tomato. The highest genome similarity was observed between phage KΦ1 and the *Xanthomonas oryzae* pv. *oryzae* specific phage OP2. On the other hand, when compared with other members of the genus *Bcep78virus*, the genome similarity was lower. Forty-four (67%) predicted KΦ1 proteins shared homology with *Xanthomonas* phage OP2, while 20 genes (30%) were unique to KΦ1. Phage KΦ1, which is chloroform resistant and stable in different media and in the pH range 5-11, showed a high titer storage ability for at least 2 years at +4°C. Copper-hydroxide and copper-oxychloride reduced phage activity proportionally to the used concentrations and the exposure time. UV light was detrimental to the phage strain, but skim milk plus sucrose formulation extended its survival *in vitro*. The phages survived for at least 7 days on the surface of pepper leaves in the greenhouse, showing the ability to persist on the plant tissue without the presence of the host bacterium. Results of three repeated experiments showed that foliar applications of the unformulated KΦ1 phage suspension effectively controlled pepper bacterial spot compared to the standard treatment and the untreated control. The integration of the phage KΦ1 and copper-hydroxide treatments resulted in an increased efficacy compared to the copper-hydroxide alone.

## Introduction

Pepper (*Capsicum annuum* L.) is one of the major vegetable crops in Serbia, covering approx. 20,000 ha of open fields. Furthermore, the area in protected environment, planted with pepper, is constantly increasing (Ignjatov et al., 2012). Large areas and intensive production, both in the open field and in plastic tunnels and greenhouses, make Serbia one of the leading pepper growing countries in Europe. However, the occurrence of pepper diseases causes significant losses and occasionally limits successful production. Bacterial spot, caused by *Xanthomonas euvesicatoria* (formerly *X. campestris* pv. *vesicatoria* group A, Jones et al., 2004; Obradović et al., 2004b), is one of the most important pepper diseases in all pepper growing areas and in Serbia as well (Balaž, 1994; Obradović et al., 2000, 2001).

The limited efficacy of current disease control strategies certainly contributes to the economic importance of this disease. Cultural practices do not provide a sufficient reduction of the disease and have not been fully implemented by commercial growers. The development by the pathogen copper or streptomycin resistance (Thayer and Stall, 1961; Marco and Stall, 1983; Adaskaveg and Hine, 1985; Minsavage et al., 1990; Pernezny et al., 1995; Martin et al., 2004) and increased public concern about detrimental effects of pesticide residues initiated efforts in searching for alternatives in control of bacterial diseases.

Bacteriophages, viruses that infect bacterial cells, have regained attention as natural antimicrobial agents to fight bacterial diseases of plants. They are natural components of the biosphere, self-replicating within host cells, highly host specific and non-toxic to eukaryotic cells (Jones et al., 2007). All these attributes, including the fact that phage multiplication and storage are fairly easy and inexpensive (Greer, 2005), make them an attractive agricultural biopesticide.

Bacteriophages have been successfully used for managing several plant diseases caused by *Xanthomonas* species, including bacterial spot of peach caused by *Xanthomonas campestris* pv. *pruni* (Civerolo and Keil, 1969; Saccardi et al., 1993), walnut blight caused by *X. campestris* pv. *juglandis* (McNeil et al., 2001), geranium bacterial blight caused by *X. campestris* pv. *pelargonii* (Flaherty et al., 2001), leaf blight of onion caused by *X. axonopodis* pv. *allii* (Lang et al., 2007), tomato bacterial spot caused by *Xanthomonas* complex (Flaherty et al., 2000; Obradović et al., 2004a, 2005), and citrus canker and citrus bacterial spot caused by *X. axonopodis* pv. *citri* and *X*. *axonopodis* pv. *citrumelo*, respectively (Balogh et al., 2008).

However, the effectiveness of phages as biocontrol agents depends not only on the susceptibility of the target bacterium, but also on environmental factors that affect phage survival. In the phyllosphere, phages can be inactivated by various factors including extreme temperatures and pH, desiccation and sunlight irradiation (especially in the UV spectrum), or they can be washed off from leaf surfaces either by rain or overhead irrigation (Suttle and Chen, 1992; Iriarte et al., 2007; Jones et al., 2007; Svircev et al., 2010). Additionally, phage persistence in the phyllosphere can be reduced by exposure to certain pesticides, such as copper compounds (Iriarte et al., 2007).

Several strategies were tested in order to increase phage vitality on foliage, such as the use of protective formulations (Balogh et al., 2003; Obradović et al., 2004a; Iriarte et al., 2007), evening or early morning application (Flaherty et al., 2000; Balogh et al., 2003), and the use of carrier bacteria for phage propagation in the environment (Svircev et al., 2006). Although it was found that copper ions inactivate phages *in vitro* (Balogh et al., 2005), this negative effect can be avoided by application of copper-based pesticides at least 4 days before phage application in the field (Balogh et al., 2008). This information indicates a possible successful integration of bacteriophages and copper compounds for disease control.

Despite the growing interest in using phages in control of phytopathogenic bacteria, there is still little information on their genome organization and structure. So far, there have been nine published whole genome sequences of phages that lyse *Xanthomonas* spp. Among them, five belong to the family *Siphoviridae* (Gene Bank acc. nos. NC_019933, NC_007709, NC_009543, NC_004902, and NC_012742), two phages belong to family *Podoviridae* (acc. nos. NC_020205 and NC_001396), one phage belongs to *Myoviridae* (acc. no. NC_007710) and one phage is classified at the level of the order *Caudovirales*. All these phages infect either *Xanthomonas axonopodis* pv. *citri, Xanthomonas oryzae* pv. *oryzae*, or *Xanthomonas campestris*. Hitherto, there has not been published a phage genome for these infecting *Xanthomonas* spp. associated with pepper or tomato.

In this paper we present the genome characteristics of a phage specific to *Xanthomonas euvesicatoria* affecting pepper, isolated from the pepper rhizosphere in Serbia (Gašić, 2011). Beside the first report of its complete genome sequence, we present the effect of some environmental factors on the phage stability and its potential to control pepper bacterial spot, under controlled conditions.

## Materials and Methods

### Bacterial Strains, Bacteriophage and Culture Conditions

Bacterial strains used in this study were stored at -80°C in glycerol (30% v/v) nutrient broth (NB) (Torlak, Serbia). The strains were grown on nutrient agar (NA; Torlak, Serbia) at 28°C for 24 h prior to use. For preparation of bacterial suspensions, cultures were suspended in sterile distilled water. The concentration was adjusted to 5 × 10^8^ CFU/ml photometrically (OD_600_ = 0.3), and then diluted accordingly.

The phage KΦ1, belonging to the family *Myoviridae*, was isolated from the rhizosphere of pepper plants showing symptoms of bacterial spot, and was stored either at +4 or at -80°C (Gašić, 2011). For the phage detection and propagation, we used either semisolid nutrient agar yeast extract medium (NYA; 0.8% NB, 0.6% agar, and 0.2% yeast extract) (Balogh et al., 2003) or NB. Phage concentrations were determined by serial dilutions and a subsequent plaque assay on NYA medium, with *Xanthomonas euvesicatoria* strain KFB189 as a host, as previously described (Gašić, 2011). Briefly, 10-fold dilutions of the phage suspension were prepared and 100 μl of each was mixed with 100 μl of the host bacterium suspension (approx. 10^9^ CFU/ml) at the bottom of a Petri dish. The NYA medium (cooled to 48°C) was poured into plates, followed by gently swirling to evenly distribute the bacteria and the phages within the medium. The plates were incubated at 27°C for 24 h to assess plaque formation. Phage concentration was estimated according to the formula for bacterial enumeration (Klement et al., 1990) and was expressed as “plaque forming units per ml” (PFU/ml).

### Determination of Phage KΦ1 Host Range

Host range analysis of phage KΦ1 was performed using 19 strains of *X. euvesicatoria*, isolated during 2015 from different localities in Serbia, including the reference strain NCPPB 2968 (**Table 1**). Additionally, we studied the phage potential lytic activity to some closely related pepper- and tomato-associated xanthomonads: *X. vesicatoria, X. gardneri*, and *X. perforans*. Besides *Xanthomonas* spp., we tested host specificity of the phage KΦ1 to some less related bacteria as well (**Table 1**). The host range test was performed by spotting 4 μl phage suspension (conc. 10^8^ PFU/ml) on the solidified NYA medium inoculated with 100 μl water suspension (conc. 10^9^ CFU/ml) of each bacterial strain. Phage activity was scored based on the spot-like clearing of the bacterial growth indicating host cell lysis. The spots were categorized as clear or turbid, indicating high host sensitivity or partial lysis, respectively. The absence of spot formation indicated a non-host relationship. The host range experiment was performed in triplicate.

**Table 1:** The host range of bacteriophage KΦ1.

Bacterial species	Strain	Origin, host, year of isolation	Source	KΦ1 phage spot formation
*Xanthomonas euvesicatoria*	KBI 116, KBI 117, KBI 118, KBI 119, KBI 120, KBI 121, KBI 123, KBI 124, KBI 125, KBI 126, KBI 127, KBI 128, KBI 129, KBI 130, KBI 131, KBI 132, KBI 133, and KBI 134	Serbia, *Capsicum annuum*, 2015	KBI^a^	+
*Xanthomonas euvesicatoria*	NCPPB 2968	United States, *Capsicum frutescens*, 1977	NCPPB^b^	+
*Xanthomonas vesicatoria*	NCPPB 1423	Hungary, *Lycopersicon esculentum*, 1957	NCPPB	–
*Xanthomonas gardneri*	NCPPB 4321	Serbia, *Lycopersicon esculentum*, 1953	NCPPB	–
*Xanthomonas perforans*	NCPPB 881	United States, *Lycopersicon esculentum*, 1991	NCPPB	–
*Acidovorax citrulli*	NCPPB 3679	United States, *Citrullus lanatus*, year unknown	NCPPB	–
*Erwinia amylovora*	KBI 32	Serbia, *Cydonia oblonga*, 2013	KBI	–
	KBI 68	Serbia, *Pyrus communis*, 2014	KBI	–
	KFB 687	Serbia, *Malus domestica*, 2013	KBI	–
	CFBP 1430	France, *Pyrus communis*, 2010	CFBP^c^	–
*Pectobacterium carotovorum* ssp. *carotovorum*	KFB 68	Serbia, *Brassica oleracea* var. *capitata*, 1999	KFB^d^	–
	KFB 85	Serbia, *Apium graveolens*, 1998	KFB	–
*Dickeya* spp.	KBI 05	United Kingdom, *Solanum tuberosum*, year unknown	KBI	–
*Ralstonia solanacearum*	NCPPB 4156	The Nederlands, *Solanum tuberosum*, 1995	NCPPB	
*Agrobacterium tumefaciens*	C58	United States, *Prunus cerasus*, 1958	S. Süle	
*Clavibacter michiganensis* ssp. *michiganensis*,	CFBP 4999	Hungary, *Lycopersicon esculentum*, 1957	CFBP	–
*Clavibacter michiganensis* ssp. *sepedonicus*	CFBP 3561	Finland, *Solanum tuberosum*, 1983	CFBP	–
*Pseudomonas syringae* pv. *lachrymans*	KFB 214	Serbia, *Cucumis sativus*, 2007	KFB	–
*Pseudomonas syringae* pv. *syringae*,	GSPB 1142	Germany, *Phaseolus* sp., 1967	GSPB^e^	–
*Pseudomonas fruorescens*^f^	B130	Ji et al. (1996)	AU	–

*+ lysis of bacterial cells (spot formation), - lack of bacterial cell lysis (no spot formation). ^*a*^KBI – Collection of Bacteria, Institute for Plant Protection and Environment, Belgrade, Serbia. ^*b*^NCPPB – National Collection of Plant Pathogenic Bacteria, United Kingdom. ^*c*^CFBP – Collection Française de Bactéries Phytopathogènes, INRA, Angers, France. ^*d*^KFB – Collection of Phytopathogenic Bacteria, University of Belgrade, Faculty of Agriculture, Serbia. ^*e*^GSPB – Göttingen Collection (Sammlung) of Phytopathogenic Bacteria, Göttingen, Germany. ^*f*^Originated from J. Kloepper, Auburn University (AU) originally designated as 89B61.*

### Bacteriophage Genomic DNA Purification

In order to obtain high phage titer for genomic DNA purification, KΦ1 phage was propagated in *X. euvesicatria* strain KFB 189 as previously described (Gašić, 2011). Following the 16 h incubation, bacterial cells were removed by centrifugation (8,000 *g* for 20 min) and the resulting phage suspension (40 ml) was filtered through a 0.22 μm membrane filter. Ten milliliter of the phage suspension was further concentrated by centrifugation (16,000 *g*, 90 min, 4°C), resuspending the phage containing pellet in 700 μl of SM buffer. The resulting phage suspension was further treated with 10 μl DNase I (1 U/μl) and 1 μl RNase A (100 mg/ml) at 37°C for 45 min to digest any bacterial nucleic acids. Phage genomic DNA was extracted by organic extraction as described by Lehman et al. (2009). DNA concentration and purity were verified using the NanoPhotometerTM Pearl (Implen GmbH, Germany). DNA integrity and absence of degradation were verified by electrophoresis on 0.8% agarose gel.

### Bacteriophage Genome Sequencing and Analysis

Phage KΦ1 genomic DNA was sequenced using the Illumina HiSeq 2500 paired-end technology at Baseclear^1^, Leiden, Netherlands following the manufacturer’s instructions. The assembly was performed using the CLC Genomics Workbench version 8.0. The IGS Annotation Engine (Institute for Genome Sciences, University of Maryland School of Medicine automated pipeline^2^) and RAST (Rapid Annotation using Subsystem Technology^3^) were used for structural and functional annotation of the sequence. The phage genome was mapped and annotated using available phage genome sequences deposited in GenBank^4^. The analysis of the genome was done using Manatee^5^ accessed via the website of the Institute for Genome Sciences, University of Maryland School of Medicine. For prediction of the phage KΦ1 lifestyle, PHACTS was used (McNair et al., 2012). In order to find potential genes coding for toxins and allergens or genes related to virulence, KΦ1 phage genome was analyzed using VirulentPred^6^ (Garg and Gupta, 2008). ARAGORN^7^ (Laslett and Canback, 2004) and tRNAscan-SE^8^ (Lowe and Chan, 2016) were used to search for tRNA genes. Genomic comparisons were performed with Mauve (Darling et al., 2010), using a progressive alignment with the default settings, and comparisons at the proteomic level were done using CoreGenes 3.5 (Zafar et al., 2002).

### Nucleotide Sequence Accession Number

The KΦ1 phage genome sequence has been deposited in the NCBI GenBank database under the accession number KY210139.

### Phage Survival in Different Media

In order to determine optimal media for the phage storage and survival, we studied the stability of phage KΦ1 in NB, tap water, distilled water, 10 mM magnesium-sulfate solution and SM buffer (10 mM Tris–HCl, pH 7.5; 100 mM NaCl; and 10 mM MgSO_4_). The media were used to prepare the phage suspension, respectively (conc. 10^10^ CFU/ml) and store it at 4°C in dark conditions. The phage concentration in each medium was checked seven times during 3 weeks, by using *X. euvesicatoria* strain KFB189 as the host, by the procedure described above. The experiment was repeated three times and the result was reported as the mean number of plaques counted (PFU/ml) for each substrate.

### Phage Survival at +4 and +20°C

Two flasks, each containing 50 ml of NB KΦ1 phage suspension (conc. 10^7^ PFU/ml), were incubated either at +4 or +20°C, in dark conditions for 6 months. Phage titer was checked at different time intervals. The plaque count assays were performed in triplicate and the results were reported as the mean number of plaques counted (PFU/ml).

### Effect of pH on Phage Viability

In order to study the effect of different pH values on phage viability, phage KΦ1 was suspended in 1 ml SM buffer, previously adjusted to pH’s 2, 5, 7, 9, 11, and 12. Phage suspensions in SM buffer were adjusted to a final concentration of 10^4^ PFU/ml. After 24 h of incubation in dark conditions at room temperature, 10-fold dilutions of each sample were prepared and assayed for phage activity. The assays were carried out in duplicate and the results were reported as the mean number of plaques counted (PFU/ml).

### Effect of UV Light on Phage Survival *in vitro*

The effect of UV light irradiation on KΦ1 phage survival was studied using two microtiter plates treated with 10% skim milk and dried in a flow hood for 3 h according to the slightly modified method of Iriarte et al. (2007). Two treatments were carried out on each plate: 24 wells per treatment were treated with 30 μl of the phage suspension (conc. 10^7^ PFU/ml) either formulated (0.75% skim milk plus 0.5% sucrose) or non-formulated. The plates were placed inside Petri dishes (R-18 cm) and stored at room temperature either in the continuous dark or exposed to UV light 254/366 nm/dark (16 h/8 h) conditions. The phage’s titer was assessed on the first day and later at different time intervals during 2 months. Three wells per treatment were assayed by adding 300 μl of tap water to each well. After 1 min, rinsates from each well were transferred into microcentrifuge tubes and titer was calculated as already described. The assays were carried out in duplicate and the results were reported as the mean number of plaques counted (PFU/ml).

### Effect of Copper Compounds on Phage Survival *in vitro*

In order to study potential negative effects of some pesticides on KΦ1 phage survival, phage suspension was mixed with commercial preparations of copper-hydroxide (Kocide 2000, DuPont) and copper-oxychloride (Bakarni oksihlorid 50, Galenika-Fitofarmacija) dissolved in tap water. Two concentrations of both copper-hydroxide (0.2 and 2%) and copper-oxychloride (0.5 and 5%) were used: the first one was determined according to the manufacturer’s recommendation for the plant treatment and the second was 10-fold higher. The KΦ1 phage suspension was added to 100 ml of each chemical to the final concentration 10^7^ PFU/ml and incubated at 20°C in dark conditions. The results were recorded on the first day and later on at a 7-day interval, during 3 weeks. The phage titer was determined as described previously and expressed as PFU/ml.

### Phage Epiphytic Survival in Greenhouse Conditions

The changes in KΦ1 phage population on plant leaves were assessed in greenhouse conditions as described by Balogh et al. (2005) with slight modifications. Pepper plants, at the 10-leaf stage, were sprayed with suspension of phage KΦ1 (conc. 10^8^ PFU/ml) in the evening (7 PM) using a handheld sprayer. The phage population was monitored by sampling fully developed terminal pepper leaves in the morning during 7 days. Each sample consisted of eight terminal leaves collected from the three plants, respectively, and placed into a sterile flask. Each sample was weighed before adding 100 ml of tap water and shaken for 30 min at 400 rpm on a horizontal shaker. An amount of 1.5 ml of rinsate was transferred to microcentrifuge tubes and treated with chloroform (1:10 v/v) for 30 min. Following incubation, the chloroform was pelleted by a pulse spin and 1 ml of the supernatant was centrifuged at 10,000 *g* for 15 min in order to remove debris. Phage concentration in the resulting suspension was calculated and expressed as the number of PFU per gram of leaf tissue by the following equation: *y* = plaque number × 1,000/dilution ratio/weight of sample (g).

### Efficacy of Bacteriophage KΦ1 in Control of Pepper Bacterial Spot in Greenhouse Conditions

High titer of phage KΦ1 (10^9^ to 10^10^ PFU/ml) was produced by inoculating log phase culture of *X. euvesicatoria* strain KFB 189 (approx. 10^8^ CFU/ml) in NB with a multiplicity of infection of 0.1, followed by overnight incubation at 28°C. Phage suspension was treated with 10% (v/v) chloroform and stored at 4°C until use. Phage concentration was diluted to 10^8^ PFU/ml just before treatment application.

*Xanthomonas euvesicatoria* strain KFB 189, sensitive to copper compounds, was used for inoculation of pepper plants. The 24-h-old culture, grown on NA, was suspended in sterile distilled water, and the concentration was adjusted to approx. 5 × 10^8^ CFU/ml photometrically (OD_600_ = 0.3), and then diluted appropriately. In the experiments 1 and 2, the concentration of inoculum was 10^8^ CFU/ml, while in the experiment 3 it was 10^6^ CFU/ml. Pepper plants of cv. Šorokšari (Institute of Vegetable Crops, Smederevska Palanka, Serbia) were grown in pots (*R* = 10 cm) containing Floradur B Fine soil (Floragard, Germany) in the greenhouse at 24–28°C. Plants were watered daily and fertilized with a soluble 18-18-18 NPK fertilizer (Navarsol IV, Timac Agro Italia) until they reached the four-leaf stage.

The treatments included single phage applications either 2 h before or 15 min after inoculation, as well as double application 2 h before and 15 min after inoculation. Integrated control was attempted by the application of copper-hydroxide (Kocide 2000, DuPont – active ingredient 53.8% copper-hydroxide) 24 h and phage 2 h before pepper inoculation. Copper-hydroxide treatment alone was used as a standard, while inoculated but untreated plants were used as a negative control. Application of treatments and inoculation were performed using a handheld sprayer. Following inoculation, plants were covered by translucent plastic bags for 48 h and arranged in a completely randomized block design in the greenhouse at 28°C.

Plants were assessed for disease severity 7 and 14 days after inoculation. The number of lesions on five leaves of each plant was evaluated. The data were analyzed by applying one-way analysis of variance (ANOVA) and Duncan’s multiple range test using Statistica for Windows statistical software (Release 7.0; StatSoft Inc., Tulsa, OK, United States).

## Results

### Host Range Analysis

Bacteriophage strain KΦ1 showed antibacterial activity against all tested *X. euvesicatoria* strains, by forming clear spots on bacterial lawns of each strain, respectively. There was not any antibacterial activity on the other tested xanthomonads affecting pepper or tomato, nor on the rest of the bacterial species used in this study (**Table 1**).

### Genome Properties

**FIGURE 1:**
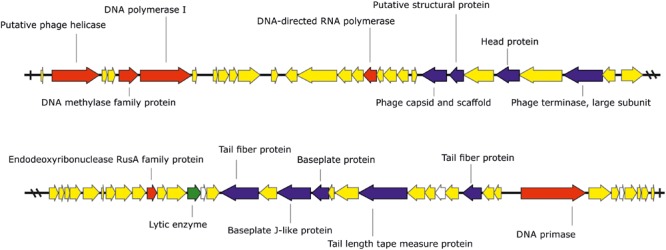
The genome of the bacteriophage KΦ1 (46,077 bp). ORFs coding for proteins involved in DNA metabolism, transcription, and translation are marked in *red*, ORFs coding for proteins involved in phage particle assembly are marked in *blue*, and ORF coding for enzyme involved in host lysis is marked in *green*. ORFs coding for hypothetical and conserved hypothetical proteins are marked in *yellow*, while ORFs of unknown function are marked in *white*. *Arrows* indicate the direction of transcription and translation. The figure was generated using the genome visualization program SnapGene http://www.snapgene.com/ ver. 2.3.4.

The phage KΦ1 has a double-stranded 46,077 bp DNA genome with GC content of 62.9% and 66 predicted open reading frames (ORFs). The average gene length was predicted to be 632 nucleotides, and 90.6% of the genome consisted of coding regions. Out of 66 putative ORFs, 16 (24.2 %) had an assigned function, three (4.5%) ORFs had unknown function, whereas the rest of 47 ORFs were classified as hypothetical (conserved) proteins (**Figure 1** and **Supplementary Table S1**). A total of six ORFs were annotated to encode proteins involved in the nucleic acid metabolism, transcription and translation (phage helicase, DNA polymerase I, DNA-directed RNA polymerase, DNA primase, DNA methylase family protein, and endodeoxyribonuclease RusA family protein). Nine ORFs were predicted to code for proteins involved in DNA packaging (phage terminase, large subunit) or virion morphogenesis (phage capsid and scaffold, putative structural protein, head protein, tail fiber proteins, baseplate J-like protein, baseplate protein, and tail length tape measure protein). One putative ORF associated with host lysis was also identified. (**Figure 1** and **Supplementary Table S1**). The genome of phage KΦ1 did not encode any transport RNAs. The start codon for transcription was ATG in 80.3% of genes, and GTG in 19.7% of phage genes.

**FIGURE 2:**
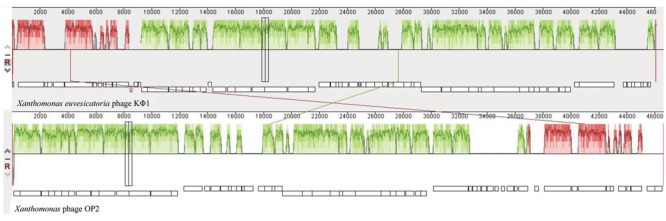
The alignment of the annotated phage KΦ1 and *Xanthomonas* phage OP2 genome sequences using Mauve. Annotated genes are shown as *white boxes*, with genes transcribed from the reverse strand shifted downward. Numbers above boxes correspond to the ORFs in KΦ1 phage (**Supplementary Table S1**) and *Xanthomonas* phage OP2 (Gene Bank acc. no. NC_007710) genomes, respectively. The degree of sequence similarity between aligned regions is indicated by the height of the similarity profile. *Connection lines* indicate similar regions between phages KΦ1 and OP2.

Comparative genomic analysis revealed that phage KΦ1 showed significant similarity only to the *Xanthomonas* phage OP2, specific to *Xanthomonas oryzae* pv. *oryzae*, a member of the genus *Bcep78virus* (ICTV, 2017). Forty-four (67%) predicted KΦ1 proteins shared homology with *Xanthomonas* phage OP2, while 20 genes (30%) were unique to KΦ1 (**Figure 2** and **Supplementary Table S1**). Lifestyle prediction by PHACTS indicated that KΦ1 was a lytic bacteriophage, which is in accordance with the observed morphology of plaques. There were no toxin genes, or genes related to virulence found in the phage genome, indicting its suitability for phage therapy.

### Phage Survival in Different Media

**FIGURE 3:**
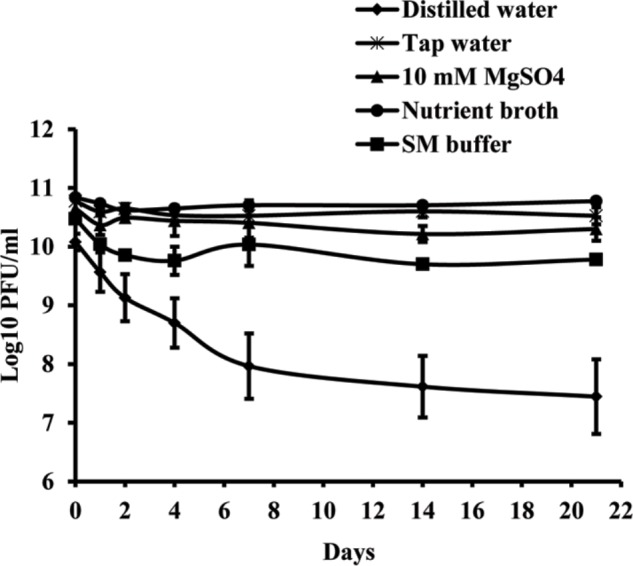
KΦ1 phage survival in different media during 3 weeks. Error bars indicate the standard error.

Studies of phage vitality in different media showed that NB was the most favorable medium for phage survival, in which the concentration of phages decreased only by 0.06 log units during 3 weeks (**Figure 3**). A decrease of phage concentration in sterile tap water, 10 mM magnesium sulfate solution and SM buffer was 0.25, 0.35, and 0.7 log units, respectively. A reduction in phage concentration was observed in the first days of experiment, while in the next days the concentration was not significantly changed. Concentration of phage KΦ1 in sterile distilled water was reduced by 2.64 log units during 3 weeks, which makes distilled water a less favorable environment for the phage survival.

### Phage Survival at +4 and +20°C

**FIGURE 4:**
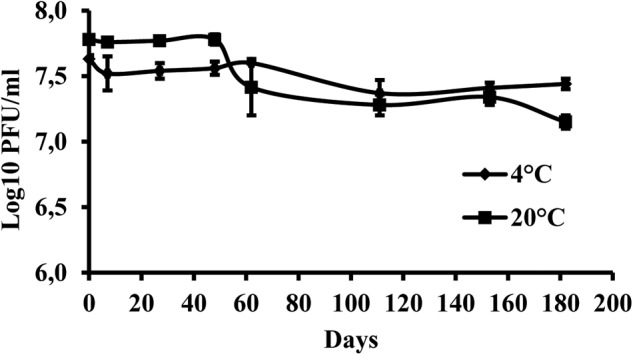
Changes in KΦ1 phage concentration at temperatures of +4 and +20°C, during 6 months of incubation. Error bars indicate the standard error.

The results indicate that the storage of the phage at +4°C provided higher stability of virus particles compared with the storage at +20°C. During the 6 months of the experiment, the initial phage concentration decreased by 0.19 log units at +4°C and by 0.63 log units at +20°C (**Figure 4**).

### Effect of pH on Phage Viability

**FIGURE 5:**
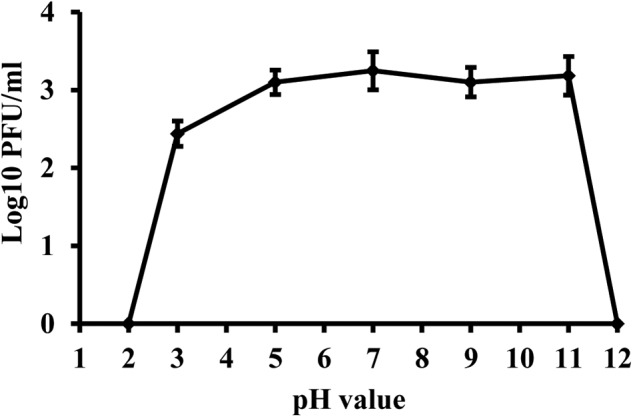
Stability of phage KΦ1 at different pH values during 24 h. Error bars indicate the standard error.

Phage KΦ1 was stable in SM buffer of pH values ranging from 5 to 11, during 24 h at room temperature and in dark conditions (**Figure 5**). The phages were completely inactivated at pH values 2 and 12 during 24 h.

### Effect of UV Light on Phage Vitality *in vitro*

**FIGURE 6:**
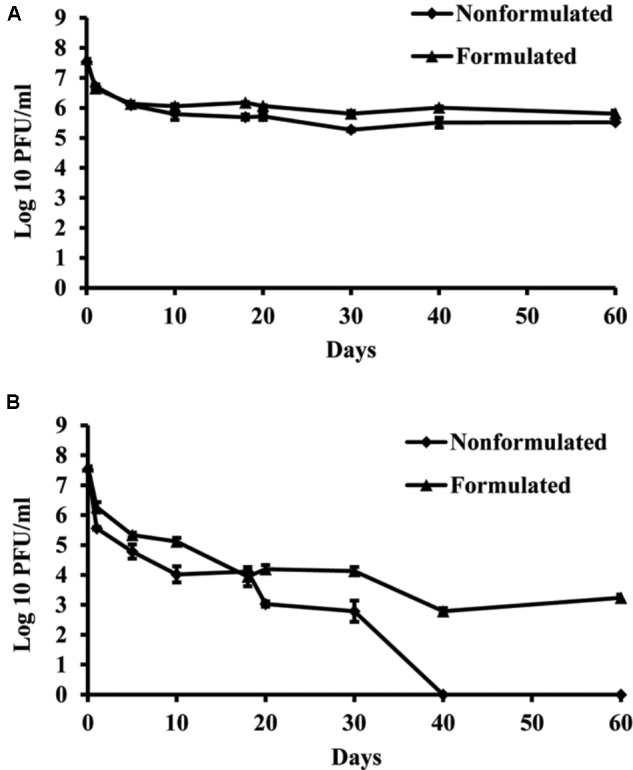
The effect of UV light on survival of KΦ1 phage. Non-formulated and formulated (0.75% skim milk plus 0.5% sucrose) phages were stored during 2 months either in completely dark conditions **(A)** or were subjected to a 16 h UV light/8 h dark photoperiod **(B)**. Error bars indicate the standard error.

Concentration of both formulated and non-formulated phages did not change significantly over the period of 60 days in dark conditions. Phage concentration decreased by 2.01 and 1.80 log units for the non-formulated and formulated phages, respectively (**Figure 6**). However, when phages were subjected to a 16 h UV light/8 h dark photoperiod, their population was reduced significantly compared to the initial concentration. The non-formulated phage population dropped to an undetectable level after 40 days of incubation. Formulation of phages provided the protective effect and influenced phage survival *in vitro* under UV/dark conditions. The formulated phage population dropped by 4.63 log units within 60 days.

### Effect of Copper Pesticides on Phage Vitality *in vitro*

**FIGURE 7:**
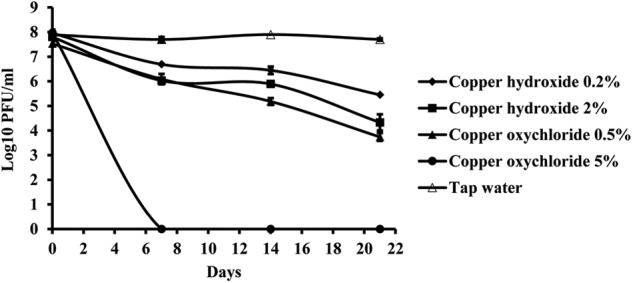
The effect of copper compounds on phage KΦ1 survival during 3 weeks. Error bars indicate the standard error.

Copper compounds reduced phage activity compared to the control, during 3 weeks. A toxic effect was observed in both application rates, the one recommended by the manufacturer as well as with a concentration ten times higher. The least toxic was copper-hydroxide in the concentration of 0.2%, which is concentration of the active ingredient in the commercial product (**Figure 7**). However, the most toxic was copper-oxychloride. Its toxicity corresponded to the concentration applied. The lower concentration (0.5%) of this compound was more toxic than the higher copper-hydroxide (2%) concentration.

### Phage Survival in Pepper Phyllosphere

**FIGURE 8:**
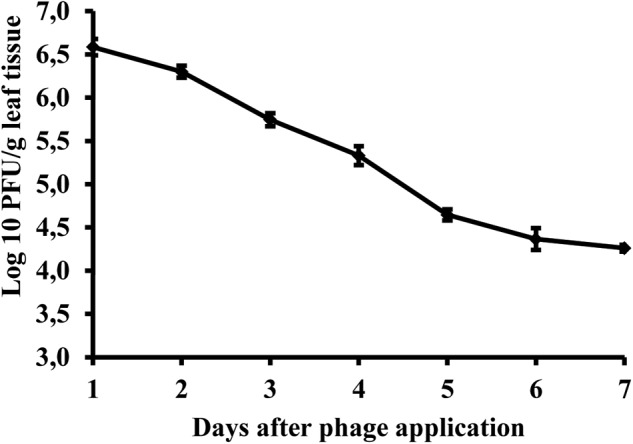
Changes in bacteriophage KΦ1 populations on pepper foliage during 7 days in greenhouse conditions. Error bars indicate the standard error.

Studies of bacteriophage KΦ1 population dynamics on pepper leaves showed that phages can persist for at least 7 days on the leaf surface in the absence of a host, under greenhouse conditions. During this period, the number of phage particles per gram leaf tissue dropped by 2.41 log units from initial concentration (**Figure 8**).

### Efficacy of Phage KΦ1 in Control of Pepper Bacterial Spot in Greenhouse Conditions

In all the experiments, the application of KΦ1 phage treatments significantly reduced the lesion number on pepper leaves compared to the untreated control (**Table 2**). The double application of phages, pre- and post-inoculation, was the most effective among the three variants of the phage treatments, but not always statistically different from the single applications. In all the experiments, there was no significant difference between the phage application 2 h before or 15 min after inoculation. However, the most efficient treatment was the integrated application of the phage suspension and copper-hydroxide. Although there was no significant difference in efficacy between this integrated application and the copper-hydroxide treatment alone, the phage application contributed to the additional reduction in the number of lesions in all the experiments (**Table 2**).

**Table 2:** The effect of phage KΦ1 treatment in pepper bacterial spot development in greenhouse conditions.

Treatments	Application timing	Average lesion number^y^		
		Experiment 1^x^	Experiment 2	Experiment 3
Phage KΦ1	2 h before inoculation	237 b	302 bc	280 b
Phage KΦ1	2 h before and 15 min after inoculation	157 cb	213 c	182 bc
Phage KΦ1	15 min after inoculation	229 b	358 ab	294 b
Copper-hydroxide^∗^ + phage KΦ1	24 h before inoculation; 2 h before inoculation	63 c	41 d	66c
Copper-hydroxide	24 h before inoculation	111 c	106 d	179 bc
Untreated control	None	332 a	422 a	567 a

*^*x*^Concentration of inoculum was 10^*8*^ CFU/ml in experiments 1 and 2 and 10^*6*^ CFU/ml in experiment 3. ^*y*^Average lesion number per plant 14 days after inoculation. Means followed by different letters (a, b, c, d) within a column are significantly different according to Duncan’s multiple range test, *P* = 0.05 level. ^∗^Kocide 2000, DuPont – active ingredient 53.8% copper-hydroxide. Concentration 0.2%, as recommended by manufacturer was used in all experiments.*

## Discussion

According to recent publications, bacteriophages have regained researchers’ attention as potential biocontrol agents in plant protection (Jones et al., 2007; Buttimer et al., 2017). Frequent occurrence of plant pathogenic bacteria, ineffective chemical control and increasing environmental concerns certainly have contributed to the increased interest in using bacteriophages for the control of bacterial infections in plants. In this research, we studied the host range, genome characteristics, survival, and biocontrol potential of bacteriophage strain KΦ1 specific to *X. euvesicatoria*, a causal agent of pepper bacterial spot.

Phage KΦ1, isolated from the pepper plant’s rhizosphere, is a lytic phage, producing clear spots on the bacterial lawn of different strains of *X. euvesicatoria*. Our previous results have shown that phage KΦ1 belongs to the *Myoviridae* family, A1 morphotype, with a latent period of 20 min and the burst size of 75 ± 4 viruses per infected cell (Gašić, 2011). Since we used chloroform during phage isolation to eliminate bacterial cells, we can confirm that KΦ1 was not sensitive to this chemical. Moreover, after 24-month storage of phage KΦ1 suspension in NB containing 10% (v/v) of chloroform, the titer was not significantly changed (unpublished data). Chloroform resistance is an important factor, since the phages which are not easily cultured and maintained, or cannot be stored on a large scale, are not desirable as potential biological control agents for their use in the field (Schisler and Slininger, 1997).

Results of phage KΦ1 host range performed during current research, including the previous study (Gašić, 2011), revealed that phage KΦ1 infected only *X. euvesicatoria* strains, but not other *Xanthomonas* spp., nor *Erwinia amylovora, Pectobacterium carotovorum* subsp. *carotovorum, Ralstonia solanacearum, Acidovorax citrulli, Clavibacter michiganensis* subsp. *sepedonicus, Clavibacter michiganensis* subsp. *michiganensis, Agrobacterium tumefaciens, Dickeya* spp., *Pseudomonas syringae* pv. *syringae*, and *Pseudomonas syringae* pv. *lachrymans*, including the saprophytic strain *P. fluorescens.* High specificity enables the elimination of target bacteria without damaging other and possibly beneficial bacteria. Moreover, the fact that phage KΦ1 has a broad host range among *X. euvesicatoria* strains is very important for its use in biocontrol of pepper bacterial spot.

The phage genome contains a dsDNA of 46,077 bp, including 66 ORFs and an average GC content 62.9%. Based on bioinformatics analysis, the closest relative of phage KΦ1 is *Xanthomonas* phage OP2 (acc. no. NC_007710) belonging to the *Myoviridae* family, genus *Bcep78virus* (Inoue et al., 2006; ICTV, 2017). OP2 is bacteriophage that lyses *Xanthomonas oryzae* pv. *oryzae*, possesses dsDNA of 46,643 bp, and the average GC content of 60.9%. For most of the 44 proteins (67%) shared by KΦ1 and OP2, the BLASTP alignments showed 43 to 84% sequence identity. Among the shared proteins, 33 of them having additional hits beside OP2, showed homology with some of the *Burkholderia cepacia* phages within the genus *Bcep78virus* (Summer et al., 2006). However, the complementary OP2 protein was the best match in all, except four cases (**Supplementary Table S1**).

Unlike some structural protein-encoding genes of the phage KΦ1, putative genes KΦ1_45 and KΦ1_51 encoding tail fiber proteins, exhibited low degree of similarity with corresponding genes of phages OP2 and/or BcepNY3 at the amino acid level (**Supplementary Table S1**). The protein sequence comparisons revealed that tail fiber protein coded by gene KΦ1_45 displayed only 53 and 55% of identity with fiber proteins of BcepNY3 and OP2 phages, respectively. A second tail fiber protein coded by gene 51 of phage KΦ1_showed 61% of identity with corresponding protein of OP2 phage. In this respect, some previous studies reported that tail fiber proteins can be involved in host specificity of the phages (Tétart et al., 1996; Le et al., 2013). Therefore, a lack of homology between tail fiber protein sequences of KΦ1 and OP2 might suggest differences in the host range of these two phages.

A study of Lavigne et al. (2008) has proposed that phages sharing at least 40% homologs or orthologs proteins belong to the same genus. Proteomic comparison of phage KΦ1 and OP2 by using CoreGenes revealed that two phages shared 60% of their proteomes. This result is consistent with the protein-by-protein comparison using BLASTP and confirms that KΦ1 and OP2 could be classified into the same genus, *Bcep78virus.* The genomes of these phages ranged from 46 to 49 kb in size and encoded 66 to 71 proteins (Lavigne et al., 2009). Furthermore, toxin genes or virulence genes were not detected, indicating that strain KΦ1 is suitable for phage therapy. The lytic lifestyle of KΦ1 phage was predicted with PHACTS as well. To our knowledge, this is the first genome sequence reported for a phage infecting *X. euvesicatoria*. This information provides a solid base for the future, advanced molecular research interaction between KΦ1 phage and its *X. euvesicatoria* host.

In order to determine the most favorable conditions for phage storage, KΦ1 phage survival in different media was studied. NB was found as the most favorable media for phage storage, followed by sterile tap water, 10 mM magnesium sulfate solution and SM buffer. In all these media, phage titter was slightly changed during 3 weeks. On the contrary, phage concentration in sterile distilled water significantly decreased during 3 weeks. Similar results were reported by Appunu and Dhar (2008), who studied the stability of phage in distilled water, saline solution and YM broth. After 30 days at 4°C in distilled water, there were no viable phage particles, whereas in other media titer declined much more slowly. The differences in phage stability in media may be due to the interaction of phage coat proteins with cations, anions and organic molecules present in the medium (Appunu and Dhar, 2008). Temperature is an important factor affecting phage stability. KΦ1 phage was stored at +4 and +20°C in NB as an optimal medium for 6 months. At the temperature of +20°C, KΦ1 phage concentration decreased by 0.44 log units more than the concentration of phage stored at 4°C, indicating that the lower temperature was more favorable for phage storage, as it was also observed by Ritchie and Klos (1979).

The negative effects of UV light on microorganisms has been widely known and investigated in various microbial ecology studies (Newsham et al., 1997; Paul et al., 1997). In our experiments, the detrimental effect of UV light particularly affected non-formulated KΦ1 phages that were completely inactivated after 40 days, while in conditions of constant darkness, phages survived more than 60 days. Skim milk and sucrose formulation significantly contributed to phage survival, especially in UV light/dark conditions. Similar results were obtained by Iriarte et al. (2007), where non-formulated phages were completely eliminated after 15 days under UV light/dark conditions, while the concentration of formulated phages decreased by 1.62 log units within 60 days. The formulation containing skim milk and sucrose developed by Balogh et al. (2003) effectively protects the phage particles from the negative effect of UV light and other environmental factors associated with survival on leaf surface. The high level of protein and sugar in milk has beneficial effects on survival of the viruses (Iriarte et al., 2007). The first information about the beneficial properties of milk for the survival of phage with the use of whey filtrate dates back to 1953 (Prouty, 1953). Also, it was reported that dextrose and tryptone can be used to protect phage particles (Ehrlich et al., 1964; Iriarte et al., 2007).

Copper ions are toxic to all cells because they react with sulfhydryl groups of certain amino acids and cause denaturation of proteins and enzymes (Agrios, 2005). Our experiment indicated the negative effect of copper hydroxide and copper oxychloride on the phage activity *in vitro*. During 3 weeks of incubation, the phage concentration decreased in suspensions of copper compounds of recommended concentration for commercial use. It was found that the concentration of copper oxychloride recommended for commercial use (0.5%) was significantly more toxic than the recommended (0.2%) and 10× higher (2%) concentration of copper hydroxide. Similarly, the negative effect of copper compounds on phage survival *in vitro* has been observed by Balogh et al. (2005), who studied the effect of copper oxychloride on the activity of the phage during 6 days. These results showed unsuitability of using copper compounds with non-formulated phages in the same tank before treatment.

A prerequisite for the successful use of bacteriophages to control plant pathogenic bacteria is that a phage comes in contact with its host on the leaf surface. The phyllosphere is a harsh environment and phages applied to aerial tissues degrade rapidly due to exposure to high temperature, high and low pH, sunlight, pesticides, or are dislodged by rain leaching (Gill and Abedon, 2003). Determining phage persistence on the pepper leaf surface was important for their future use as biological agents in bacterial spot control. Our results showed that KΦ1 phages may persist at least 7 days on pepper leaf surface under greenhouse conditions, without the presence of the host bacterium. This finding is significant for defining the time interval of future phage treatment in disease control. However, we should keep in mind that phage persistence in the greenhouse is probably longer than its persistence in the field, since the greenhouse protects phages from the negative effects of some environmental factors. The intensity of UV light is significantly reduced due to the passage of sunlight through a glass surface. Also, greenhouse conditions protect phages from extremely high temperatures and washing of viruses by rain, and contribute to their persistence in the phyllosphere. Similar results were obtained by Zaccardelli et al. (1992), who studied the survival of phage specific to *X. campestris* pv. *pruni* on the surface of peach leaves in the absence of a host. Phages survived for 5 days on the leaf surface in a climatic chamber, where phage concentration was decreased from an initial value of 1.12 × 10^7^ to 2.6 × 10^5^ PFU/g leaf tissue. Epiphytic survival of phage on the leaves in the orchard was at least 5 days, and approximately 10^4^ times lower than its survival on the leaves in the climatic chamber. Balogh et al. (2005) recovered phages from the tomato canopy up to 2 and 4 days after application under field and greenhouse conditions, respectively. The reduction in phage populations occurred mainly during the daytime. One of the unique advantages of phages compared to chemical pesticides, however, is their ability to increase their concentration by multiplying on a bacterial host. Under favorable environmental conditions, in the presence of high host populations, phages persist much better than without the host (Balogh et al., 2009).

The efficacy of phage KΦ1 as a biological agent in control of pepper bacterial spot in greenhouse conditions was confirmed in all three experiments. All phage treatments significantly reduced the intensity of bacterial leaf spot of pepper compared to the untreated control. However, a single application of KΦ1 phages did not achieve a constant level of efficacy. Such results could be explained by the limited survival of non-formulated phages on the leaf surface. Similar inconsistency was observed by Obradović et al. (2005), where phage treatments were applied in control of tomato bacterial spot. Our results did not show any significant differences in disease reduction between the phage applications before and after inoculation. The most efficient treatment was the combination when copper compound was applied 1 day and phages 2 h before inoculation. Although it was reported that copper compounds may be detrimental to phages (Balogh et al., 2005; Iriarte et al., 2007), the copper-hydroxide application approximately 1 day before the application of phages did not affect the phage efficacy, based on the number of lesions counted (**Table 2**). However, the combination of these treatments contributed to the higher efficacy.

Biological control using bacteriophages as biological agents is a sustainable strategy in plant protection, since biopesticides are quickly degraded in the ecosystem. In addition, there is no phytotoxicity, resistant bacterial strains develop more slowly, and they are considered as safe for human health. Our results clearly show that phage KΦ1 possesses high specificity and lytic activity to a range of *X. euvesicatoria* strains. These findings, as well as other studied characteristics, make this phage a valuable candidate for biological control of bacterial spot of pepper. Current study provides the starting point for the future research in order to improve phage KΦ1 stability and efficacy in disease control in field conditions.

## Author Contributions

KG and AO conceived and designed the experiments. KG, MI, AP, and MŠ performed the experiments. KG, NK, and AO analyzed the data. KG wrote the paper. AO revised the paper. All authors read and approved the final manuscript.

## Conflict of Interest Statement

The authors declare that the research was conducted in the absence of any commercial or financial relationships that could be construed as a potential conflict of interest.

## Footnotes

1 www.baseclear.com

2 http://ae.igs.umaryland.edu/cgi/index.cgi

3 http://rast.nmpdr.org/website

4 http://www.ncbi.nlm.nih.gov/genbank/

5 http://manatee.sourceforge.net/

6 http://203.92.44.117/virulent/submit.html

7 http://130.235.46.10/ARAGORN/

8 http://lowelab.ucsc.edu/tRNAscan-SE
