# Supplementary material for: Complete Genome of the Xanthomonas euvesicatoria Specific Bacteriophage KΦ1, Its Survival and Potential in Control of Pepper Bacterial Spot
Source: Front Microbiol. 2018 Aug 29;9:2021. doi: 10.3389/fmicb.2018.02021 (PMC6123377; doi:10.3389/fmicb.2018.02021)
Supplement: Supplementary file 1 [file Table_1.docx]

Supplementary Table 1. Putative open reading frames (ORFs) of bacteriophage KΦ1 and their predicted functions.

| No. | Locus tag  (ORF) | 5' end | 3' end | Predicted function  (Pfam) | Protein length  (amino acids) | Taxon | % identity | P-value |
| --- | --- | --- | --- | --- | --- | --- | --- | --- |
| 1 | KΦ1_1 | 18 | 128 | Hypothetical protein | 36 | Unknown |  |  |
| 2 | [KΦ1_2](https://manatee.igs.umaryland.edu/tigr-scripts/chado_prok_manatee/shared/ORF_infopage.cgi?db=K1pha&orf=K1pha.transcript.11905790483.1) | 440 | 2272 | Putative phage helicase | 610 | *Xanthomonas* phage OP2 | 78.9 | 0 |
| 3 | KΦ1_3 | 2341 | 2544 | Hypothetical protein | 67 | Unknown |  |  |
| 4 | KΦ1_4 | 2544 | 2867 | Hypothetical protein | 107 | Unknown |  |  |
| 5 | [KΦ1_5](https://manatee.igs.umaryland.edu/tigr-scripts/chado_prok_manatee/shared/ORF_infopage.cgi?db=K1pha&orf=K1pha.transcript.11905790509.1) | 2968 | 3735 | DNA methylase family protein | 255 | *Burkholderia* phage Bcep43 | 51.6 | 1.0E-60 |
| 6 | [KΦ1_6](https://manatee.igs.umaryland.edu/tigr-scripts/chado_prok_manatee/shared/ORF_infopage.cgi?db=K1pha&orf=K1pha.transcript.11905790620.1) | 3772 | 5742 | DNA polymerase I | 656 | *Xanthomonas* phage OP2 | 76.3 | 0 |
| 7 | KΦ1_7 | 5745 | 5945 | Hypothetical protein | 66 | Unknown |  |  |
| 8 | KΦ1_8 | 6521 | 6712 | Hypothetical protein | 63 | Unknown |  |  |
| 9 | KΦ1_9 | 6709 | 7143 | Conserved hypothetical protein | 144 | *Xanthomonas* phage OP2 | 51.8 | 1.0E-21 |
| 10 | KΦ1_10 | 7143 | 7460 | Conserved hypothetical protein | 105 | *Xanthomonas* phage OP2 | 61.5 | 3.0E-24 |
| 11 | KΦ1_11 | 7477 | 8340 | Conserved hypothetical protein | 287 | *Xanthomonas* phage OP2 | 41.4 | 2.0E-15 |
| 12 | KΦ1_12 | 8709 | 8996 | Hypothetical protein | 95 | Unknown |  |  |
| 13 | KΦ1_13 | 9253 | 9687 | Conserved hypothetical protein | 144 | *Xanthomonas* phage OP2 | 74.3 | 2.0E-55 |
| 14 | KΦ1_14 | 9700 | 11184 | Conserved hypothetical protein | 494 | *Xanthomonas* phage OP2 | 71.7 | 1.0E-163 |
| 15 | [KΦ1_15](https://manatee.igs.umaryland.edu/tigr-scripts/chado_prok_manatee/shared/ORF_infopage.cgi?db=K1pha&orf=K1pha.transcript.11905790505.1) | 11739 | 11209 | Conserved hypothetical protein | 176 | *Xanthomonas* phage OP2 | 69.9 | 6.0E-66 |
| 16 | [KΦ1_16](https://manatee.igs.umaryland.edu/tigr-scripts/chado_prok_manatee/shared/ORF_infopage.cgi?db=K1pha&orf=K1pha.transcript.11905790720.1) | 12185 | 11739 | Conserved hypothetical protein | 149 | *Xanthomonas* phage OP2 | 53.6 | 7.0E-34 |
| 17 | [KΦ1_17](https://manatee.igs.umaryland.edu/tigr-scripts/chado_prok_manatee/shared/ORF_infopage.cgi?db=K1pha&orf=K1pha.transcript.11905790783.1) | 12691 | 12182 | DNA-directed RNA polymerase | 169 | *Xanthomonas* phage OP2 | 71.9 | 3.0E-63 |
| 18 | KΦ1_18 | 12687 | 12948 | Conserved hypothetical protein | 176 | *Xanthomonas* phage OP2 | 69.9 | 6.0E-66 |
| 19 | KΦ1_19 | 13448 | 12945 | Conserved hypothetical protein | 167 | *Xanthomonas* phage OP2 | 71.3 | 4.0E-64 |
| 20 | KΦ1_20 | 13887 | 13462 | Conserved hypothetical protein | 141 | *Xanthomonas* phage OP2 | 72.9 | 7.0E-50 |
| 21 | KΦ1_21 | 14013 | 14249 | Hypothetical protein | 78 | Unknown |  |  |
| 22 | [KΦ1_22](https://manatee.igs.umaryland.edu/tigr-scripts/chado_prok_manatee/shared/ORF_infopage.cgi?db=K1pha&orf=K1pha.transcript.11905790570.1) | 15313 | 14375 | Phage capsid and scaffold | 312 | *Xanthomonas* phage OP2 | 76.9 | 1.0E-140 |
| 23 | [KΦ1_23](https://manatee.igs.umaryland.edu/tigr-scripts/chado_prok_manatee/shared/ORF_infopage.cgi?db=K1pha&orf=K1pha.transcript.11905790799.1) | 15957 | 15424 | Putative structural protein | 177 | *Xanthomonas* phage OP2 | 76.3 | 4.0E-60 |
| 24 | KΦ1_24 | 17112 | 15973 | Conserved hypothetical protein | 379 | *Xanthomonas* phage OP2 | 71.3 | 1.0E-123 |
| 25 | [KΦ1_25](https://manatee.igs.umaryland.edu/tigr-scripts/chado_prok_manatee/shared/ORF_infopage.cgi?db=K1pha&orf=K1pha.transcript.11905790791.1) | 18072 | 17188 | Head protein | 294 | *Xanthomonas* phage OP2 | 67 | 1.0E-108 |
| 26 | KΦ1_26 | 19681 | 18047 | Conserved hypothetical protein | 544 | *Xanthomonas* phage OP2 | 74.9 | 0 |
| 27 | [KΦ1_27](https://manatee.igs.umaryland.edu/tigr-scripts/chado_prok_manatee/shared/ORF_infopage.cgi?db=K1pha&orf=K1pha.transcript.11905790761.1) | 21196 | 19730 | Phage terminase, large subunit | 488 | *Xanthomonas* phage OP2 | 83.8 | 0 |
| 28 | [KΦ1_28](https://manatee.igs.umaryland.edu/tigr-scripts/chado_prok_manatee/shared/ORF_infopage.cgi?db=K1pha&orf=K1pha.transcript.11905790515.1) | 21678 | 21193 | Conserved hypothetical protein | 161 | *Xanthomonas* phage OP2 | 78.7 | 1.0E-55 |
| 29 | [KΦ1_29](https://manatee.igs.umaryland.edu/tigr-scripts/chado_prok_manatee/shared/ORF_infopage.cgi?db=K1pha&orf=K1pha.transcript.11905790460.1) | 21918 | 22760 | Conserved hypothetical protein | 280 | *Xanthomonas* phage OP2 | 59.7 | 7.0E-73 |
| 30 | KΦ1_30 | 22819 | 23169 | Conserved hypothetical protein | 116 | *Xanthomonas* phage OP2 | 61.5 | 1.0E-39 |
| 31 | KΦ1_31 | 23169 | 23393 | Hypothetical protein | 74 | Unknown |  |  |
| 32 | KΦ1_32 | 23390 | 23623 | Hypothetical protein | 77 | Unknown |  |  |
| 33 | KΦ1_33 | 23607 | 24026 | Conserved hypothetical protein | 139 | *Xanthomonas* phage OP2 | 33.6 | 3.0E-12 |
| 34 | KΦ1_34 | 24099 | 24737 | Conserved hypothetical protein | 212 | *Xanthomonas* phage OP2 | 50.5 | 2.0E-30 |
| 35 | KΦ1_35 | 24791 | 24910 | Hypothetical protein | 39 | Unknown |  |  |
| 36 | KΦ1_36 | 24964 | 25329 | Hypothetical protein | 121 | Unknown |  |  |
| 37 | KΦ1_37 | 25322 | 25951 | Conserved hypothetical protein | 209 | Unknown |  |  |
| 38 | [KΦ1_38](https://manatee.igs.umaryland.edu/tigr-scripts/chado_prok_manatee/shared/ORF_infopage.cgi?db=K1pha&orf=K1pha.transcript.11905790709.1) | 25948 | 26490 | Hypothetical protein | 180 | *Xanthomonas* phage OP2 | 43.3 | 7.0E-22 |
| 39 | [KΦ1_39](https://manatee.igs.umaryland.edu/tigr-scripts/chado_prok_manatee/shared/ORF_infopage.cgi?db=K1pha&orf=K1pha.transcript.11905790729.1) | 26511 | 26891 | Endodeoxyribonuclease RusA family protein | 126 | *Burkholderia* phage BcepNY3 | 49.2 | 3.0E-18 |
| 40 | KΦ1_40 | 26888 | 27259 | Hypothetical protein | 123 | Unknown |  |  |
| 41 | KΦ1_41 | 27256 | 28014 | Hypothetical protein | 252 | Unknown |  |  |
| 42 | [KΦ1_42](https://manatee.igs.umaryland.edu/tigr-scripts/chado_prok_manatee/shared/ORF_infopage.cgi?db=K1pha&orf=K1pha.transcript.11905790455.1) | 28026 | 28550 | Lytic enzyme | 174 | *Xanthomonas* phage OP2 | 73.4 | 7.0E-66 |
| 43 | KΦ1_43 | 28547 | 28768 | Unknown | 73 | Unknown |  |  |
| 44 | KΦ1_44 | 28758 | 29255 | Conserved hypothetical protein | 165 | *Xanthomonas* phage OP2 | 74.7 | 8.0E-57 |
| 45 | [KΦ1_45](https://manatee.igs.umaryland.edu/tigr-scripts/chado_prok_manatee/shared/ORF_infopage.cgi?db=K1pha&orf=K1pha.transcript.11905790451.1) | 30713 | 29289 | Tail fiber protein | 474 | *Burkholderia* phage BcepNY3 | 53.2 | 9.0E-65 |
| 46 | KΦ1_46 | 30723 | 31403 | Conserved hypothetical protein | 226 | *Xanthomonas* phage OP2 | 77.1 | 1.0E-102 |
| 47 | [KΦ1_47](https://manatee.igs.umaryland.edu/tigr-scripts/chado_prok_manatee/shared/ORF_infopage.cgi?db=K1pha&orf=K1pha.transcript.11905790601.1) | 32671 | 31403 | Baseplate J-like protein | 422 | *Xanthomonas* phage OP2 | 73.5 | 1.0E-157 |
| 48 | [KΦ1_48](https://manatee.igs.umaryland.edu/tigr-scripts/chado_prok_manatee/shared/ORF_infopage.cgi?db=K1pha&orf=K1pha.transcript.11905790495.1) | 33354 | 32689 | Baseplate protein | 221 | *Xanthomonas* phage OP2 | 73.2 | 2.0E-71 |
| 49 | KΦ1_49 | 33572 | 33351 | Conserved hypothetical protein | 73 | *Xanthomonas* phage OP2 | 47.7 | 2.0E-09 |
| 50 | [KΦ1_50](https://manatee.igs.umaryland.edu/tigr-scripts/chado_prok_manatee/shared/ORF_infopage.cgi?db=K1pha&orf=K1pha.transcript.11905790704.1) | 34470 | 33574 | Conserved hypothetical protein | 298 | *Xanthomonas* phage OP2 | 64.3 | 1.0E-104 |
| 51 | [KΦ1_51](https://manatee.igs.umaryland.edu/tigr-scripts/chado_prok_manatee/shared/ORF_infopage.cgi?db=K1pha&orf=K1pha.transcript.11905790616.1) | 36302 | 34467 | Tail length tape measure protein | 611 | *Xanthomonas* phage OP2 | 53.4 | 1.0E-123 |
| 52 | [KΦ1_52](https://manatee.igs.umaryland.edu/tigr-scripts/chado_prok_manatee/shared/ORF_infopage.cgi?db=K1pha&orf=K1pha.transcript.11905790525.1) | 36971 | 36306 | Conserved hypothetical protein | 221 | *Xanthomonas* phage OP2 | 71.1 | 3.0E-76 |
| 53 | KΦ1_53 | 37373 | 36975 | Conserved hypothetical protein | 132 | *Xanthomonas* phage OP2 | 76.7 | 8.0E-51 |
| 54 | KΦ1_54 | 37747 | 37370 | Unknown | 125 | Unknown |  |  |
| 55 | [KΦ1_55](https://manatee.igs.umaryland.edu/tigr-scripts/chado_prok_manatee/shared/ORF_infopage.cgi?db=K1pha&orf=K1pha.transcript.11905790740.1) | 38259 | 37720 | Conserved hypothetical protein | 179 | *Xanthomonas* phage OP2 | 71.9 | 3.0E-56 |
| 56 | [KΦ1_56](https://manatee.igs.umaryland.edu/tigr-scripts/chado_prok_manatee/shared/ORF_infopage.cgi?db=K1pha&orf=K1pha.transcript.11905790546.1) | 39099 | 38380 | Tail fiber protein | 239 | *Xanthomonas* phage OP2 | 60.7 | 5.0E-14 |
| 57 | KΦ1_57 | 39580 | 39167 | Conserved hypothetical protein | 137 | *Xanthomonas* phage OP2 | 53.7 | 5.0E-30 |
| 58 | [KΦ1_58](https://manatee.igs.umaryland.edu/tigr-scripts/chado_prok_manatee/shared/ORF_infopage.cgi?db=K1pha&orf=K1pha.transcript.11905790724.1) | 39846 | 39577 | Conserved hypothetical protein | 89 | *Xanthomonas* phage OP2 | 73.3 | 3.0E-31 |
| 59 | [KΦ1_59](https://manatee.igs.umaryland.edu/tigr-scripts/chado_prok_manatee/shared/ORF_infopage.cgi?db=K1pha&orf=K1pha.transcript.11905790536.1) | 40601 | 43084 | DNA primase | 827 | *Burkholderia* phage BcepNY3 | 49.9 | 0 |
| 60 | KΦ1_60 | 43159 | 44013 | Hypothetical protein | 284 | Unknown |  |  |
| 61 | KΦ1_61 | 44017 | 44322 | Conserved hypothetical protein | 101 | *Xanthomonas* phage OP2 | 54.5 | 1.0E-19 |
| 62 | KΦ1_62 | 44319 | 44528 | Unknown | 69 | Unknown |  |  |
| 63 | KΦ1_63 | 44525 | 44989 | Conserved hypothetical protein | 154 | *Xanthomonas* phage OP2 | 42.9 | 5.0E-08 |
| 64 | KΦ1_64 | 44986 | 45168 | Hypothetical protein | 60 | Unknown |  |  |
| 65 | KΦ1_65 | 45165 | 45431 | Hypothetical protein | 88 | Unclassified phages | 51.6 | 5.0E-16 |
| 66 | KΦ1_66 | 45511 | 45672 | Hypothetical protein | 53 | Unknown |  |  |
